# Plectin is a novel regulator for apical extrusion of RasV12-transformed cells

**DOI:** 10.1038/srep44328

**Published:** 2017-03-10

**Authors:** Ailijiang Kadeer, Takeshi Maruyama, Mihoko Kajita, Tomoko Morita, Ayana Sasaki, Atsuko Ohoka, Susumu Ishikawa, Masaya Ikegawa, Takashi Shimada, Yasuyuki Fujita

**Affiliations:** 1 Division of Molecular Oncology, Institute for Genetic Medicine, Hokkaido University, Sapporo 060-0815, Japan; 2 Graduate School of Medicine, Hokkaido University, Sapporo 060-0815, Japan; 3 Graduate School of Chemical Sciences and Engineering, Hokkaido University, Sapporo 060-0815, Japan; 4 Genomics, Proteomics and Biomedical Functions, Department of Life and Medical Systems, Faculty of Life and Medical Sciences, Doshisha University, Kyotanabe City 610-0394, Japan; 5 SHIMADZU Corporation, Tokyo 101-8448, Japan

## Abstract

Several lines of evidence have revealed that newly emerging transformed cells are often eliminated from the epithelium, though the underlying molecular mechanisms of this cancer preventive phenomenon still remain elusive. In this study, using mammalian cell culture systems we have identified plectin, a versatile cytoskeletal linker protein, as a novel regulator for apical extrusion of RasV12-transformed cells. Plectin is accumulated in RasV12 cells when they are surrounded by normal epithelial cells. Similarly, cytoskeletal proteins tubulin, keratin, and Epithelial Protein Lost In Neoplasm (EPLIN) are also accumulated in the transformed cells surrounded by normal cells. Knockdown or functional disruption of one of these molecules diminishes the accumulation of the others, indicating that the accumulation process of the individual protein mutually depends on each other. Furthermore, plectin-knockdown attenuates caveolin-1 (Cav-1) enrichment and PKA activity in RasV12 cells and profoundly suppresses the apical extrusion. These results indicate that the plectin-microtubules-EPLIN complex positively regulates apical elimination of RasV12-transformed cells from the epithelium in a coordinated fashion. Further development of this study would open a new avenue for cancer preventive medicine.

In most of the multicellular organisms such as fly and mammals, oncogenic mutations occur within the epithelial tissues at the initial stage of carcinogenesis, though the fate of the transformed cells remained enigmatic. Recent studies by us and others, however, have revealed that the newly emerging transformed cells are often eliminated from the epithelium. During this process, normal and transformed epithelial cells compete with each other for survival, a process called cell competition^1^,^2^,^3^,^4^,^5^,^6^,^7^,^8^,^9^,^10^. For example, when Ras- or Src-mutated cells appear within the epithelial monolayer, normal cells recognize the presence of transformed cells and actively eliminate them into the apical lumen^11^,^12^; this cancer preventive mechanism is termed EDAC (Epithelial Defense Against Cancer)^13^. The apical extrusion of Ras-transformed cells involves various non-cell-autonomous changes in both normal and transformed cells. In the transformed cells, Epithelial Protein Lost In Neoplasm (EPLIN) is accumulated at the apical and lateral membrane domains, thereby regulating the downstream molecules including protein kinase A (PKA) and caveolin-1 (Cav-1), leading to apical extrusion of transformed cells^14^. In the neighboring normal epithelial cells, cytoskeletal proteins filamin and vimentin are accumulated at the interface with transformed cells, which exert physical forces that are required for apical extrusion^13^. But, to fully understand the whole puzzling picture of cell competition between normal and transformed cells, missing pieces need to be further uncovered.

Plectin is a versatile cytoskeletal linker protein of high molecular weight (>500 kDa)^15^,^16^,^17^,^18^. It binds to a number of cytoskeletal proteins including microtubules and intermediate filaments and is involved in establishment and dynamic modulation of the cytoskeletal network. In this study, we have identified plectin as a new player acting in the apical extrusion of RasV12-transformed cells.

## Results

### Plectin is a novel regulator for apical extrusion of RasV12-transformed epithelial cells

**Figure 1:**
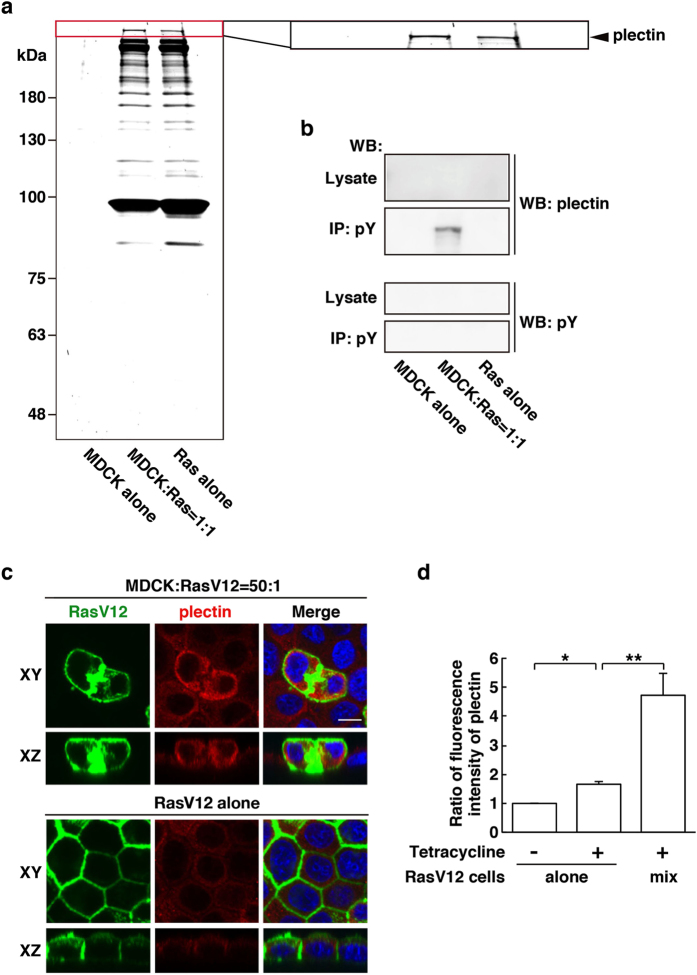
Plectin is accumulated in RasV12-transformed cells that are surrounded by normal epithelial cells. (**a**) SYPRO ruby staining (9% SDS-PAGE) of immunoprecipitated proteins with a mixture of anti-phospho-tyrosine antibodies. Cells were cultured under three different conditions: (i) normal MDCK cells alone, (ii) 1:1 mix culture of normal and RasV12-transformed MDCK cells, and (iii) RasV12-transformed MDCK cells alone. The cell lysates were collected after 16 h incubation with tetracycline, followed by immunoprecipitation. The area in the red box is shown at higher magnification in the right panel. The arrowhead indicates the band for plectin. (**b**) Validation of the band for plectin by western blotting. Immunoprecipitated samples were examined by western blotting with anti-plectin or anti-phospho-tyrosine antibody. (**c**) Immunofluorescence images of plectin in the mix culture of normal and RasV12-transformed MDCK cells. MDCK-pTR GFP-RasV12 cells were mixed with normal MDCK cells or cultured alone on collagen gels. Cells were fixed after 16 h incubation with tetracycline and stained with anti-plectin antibody (red) and Hoechst (blue). Scale bar, 10 μm. (**d**) Quantification of the fluorescence intensity of plectin. Data are mean ± SD from three independent experiments. *P < 0.0005 and **P < 0.05; n = 30 cells for each experimental condition. Values are expressed as a ratio relative to RasV12 cells alone Tet (−).

To examine the competitive interaction between normal and transformed cells, we have established Madin-Darby canine kidney (MDCK) epithelial cells stably expressing oncogenic RasV12 or cSrcY527F in a tetracycline-inducible manner^11^,^14^. Normal and tetracycline-inducible transformed MDCK cells are co-cultured in the absence of tetracycline until they form a monolayer. Then, tetracycline is added to induce expression of oncoproteins, which allows us to analyze the interaction between normal and newly emerging transformed cells. In a previous study, we found three molecules that were immunoprecipitated with anti-phospho-tyrosine antibodies specifically under the mix culture of normal and Src-transformed MDCK cells (Supplementary Fig. S1a)^13^. We then identified the 280 kDa and 55 kDa proteins as filamin A and vimentin respectively and demonstrated that they were accumulated in normal cells at the interface with transformed cells and play a positive role in apical elimination of the transformed cells^13^. Here, we first analyzed the remaining third molecule using mass spectrometry and identified the 500 kDa protein as plectin (Supplementary Fig. S1a). In addition, using tetracycline-inducible RasV12-expressing MDCK cells we demonstrated that the amount of immunoprecipitated plectin with anti-phospho-tyrosine antibodies was increased under the mix culture of normal and RasV12-transformed cells, compared with single culture of normal or RasV12-transformed cells (Fig. 1a,b). By western blotting with anti-phospho-tyrosine antibody, we could not detect tyrosine-phosphorylation of plectin *per se* (Fig. 1b), similarly to filamin and vimentin^13^, suggesting that plectin binds to unidentified, tyrosine-phosphorylated protein(s).

**Figure 2:**
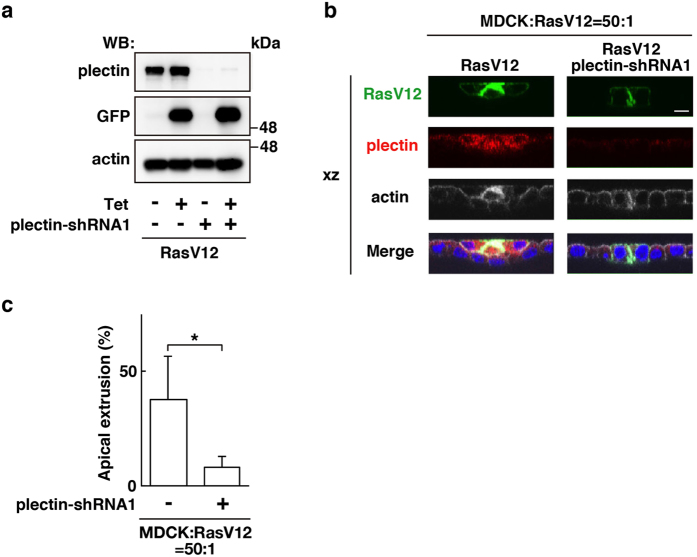
Plectin plays a positive role in apical extrusion of RasV12-transformed cells. (**a**) Establishment of MDCK-pTR GFP-RasV12 cells stably expressing plectin-shRNA1. Expression of GFP-RasV12 was induced with tetracycline treatment, followed by western blotting with the indicated antibodies. (**b**) XZ images of RasV12 cells or plectin-knockdown RasV12 cells that were surrounded by normal epithelial cells. MDCK-pTR GFP-RasV12 cells or MDCK-pTR GFP-RasV12 plectin-shRNA1 cells were mixed with normal MDCK cells. Cells were stained with anti-plectin antibody (red), Alexa-Fluor-648-phalloidin (white), and Hoechst (blue). Scale bar, 10 μm. (**c**) Quantification of the effect of plectin-knockdown on apical extrusion of RasV12 cells. Data are mean ± SD from seven and four independent experiments. *P < 0.05; n = 100–290 cells for each experimental condition.

We then examined subcellular localization of plectin by immunofluorescence analysis. When MDCK-pTR GFP-RasV12 cells were cultured alone, induction of RasV12 expression by tetracycline slightly increased immunofluorescence intensity of plectin in a cell-autonomous manner (Fig. 1d and Supplementary Fig. S1b). But, when RasV12 cells were surrounded by normal cells, accumulation of plectin was further enhanced (Fig. 1c,d and Supplementary Fig. S1b), indicating that the presence of surrounding normal cells promoted the accumulation of plectin in a non-cell-autonomous fashion. The accumulation of plectin was observed in both apically extruding cells and already extruded cells (Figs 1c and 2b), suggesting that plectin is accumulated in transformed cells during the process of apical extrusion. Similarly, plectin was accumulated in Src-transformed cells, when they were surrounded by normal cells (Supplementary Fig. S1c).

To understand the functional significance of the plectin accumulation, we established RasV12-transformed MDCK cells stably expressing plectin-shRNA (Fig. 2a and Supplementary Fig. S2a). We demonstrated that knockdown of plectin substantially suppressed apical extrusion of RasV12-transformed cells (Fig. 2b,c and Supplementary Fig. S2b), indicating that plectin plays a positive role in the apical elimination of transformed cells. Despite of several trials, we were unable to establish Src-transformed cell lines stably expressing plectin-shRNA, thus hereafter mainly focused on RasV12 cells for functional analyses.

### Microtubules and plectin mutually regulate their accumulation and promote apical extrusion of RasV12-transformed cells

**Figure 3:**
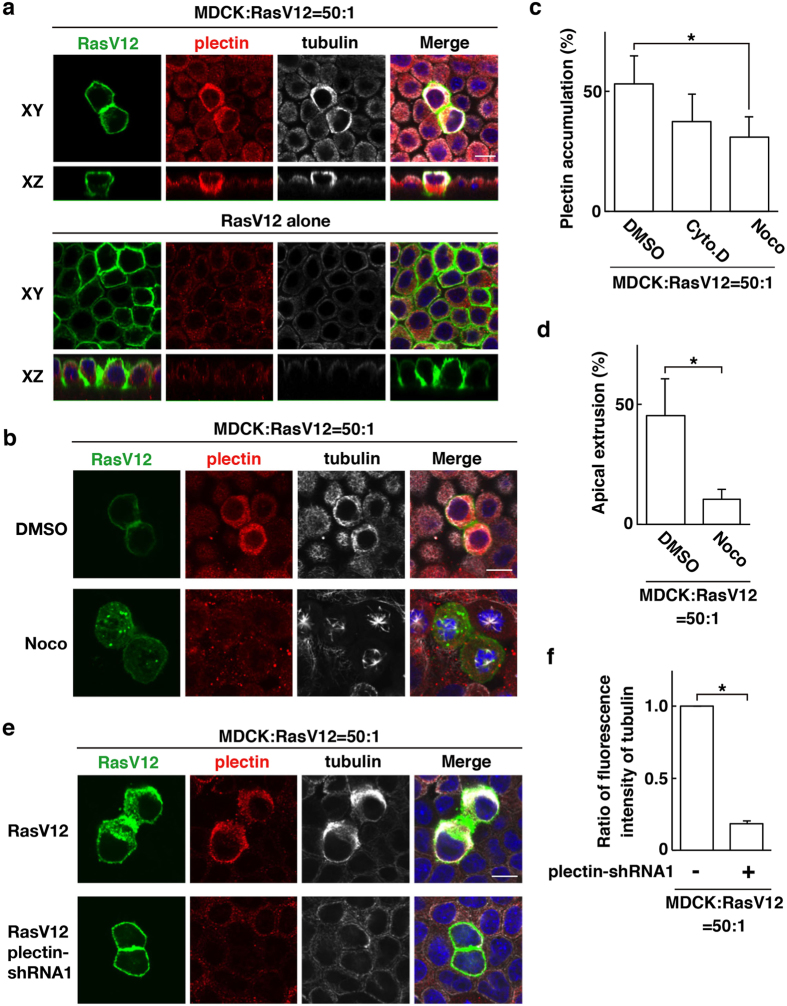
Microtubules and plectin mutually regulate their accumulation and promote apical extrusion of RasV12-transformed cells. (**a**) Accumulation of plectin and tubulin in RasV12-transformed cells surrounded by normal epithelial cells. MDCK-pTR GFP-RasV12 cells were mixed with normal MDCK cells or cultured alone on collagen gels. Cells were fixed after 16 h incubation with tetracycline and stained with anti-plectin (red) and anti-tubulin (white) antibodies and Hoechst (blue). (**b**,**c**) Effect of nocodazole or cytochalasin D on accumulation of plectin and tubulin. (**c**) Data are mean ± SD from four independent experiments. *P < 0.05; n = 140–180 cells for each experimental condition. (**d**) Effect of nocodazole on apical extrusion of RasV12-transformed cells. Data are mean ± SD from three independent experiments. *P < 0.05; n = 100–130 cells for each experimental condition. (**e**,**f**) Effect of plectin-knockdown on accumulation of tubulin. MDCK-pTR GFP-RasV12 cells or MDCK-pTR GFP-RasV12 plectin-shRNA1 cells were mixed with normal MDCK cells. (**a**,**b**,**e**) Scale bars, 10 μm. (**f**) Data are mean ± SD from three independent experiments. *P < 0.0001; n = 30 cells for each experimental condition. Values are expressed as a ratio relative to plectin-shRNA1 (−).

Plectin crosslinks microtubules and intermediate filaments and regulates their dynamics^15^,^16^,^17^,^18^. Therefore, we next examined functional relationship between plectin and those cytoskeletal proteins. By immunofluorescence, we found that tubulin, a major component of microtubules, was accumulated in the apical region of RasV12-transformed cells that were surrounded by normal cells, which was partially co-localized with plectin (Fig. 3a). When RasV12 cells alone were present, accumulation of tubulin did not occur (Fig. 3a). Comparable non-cell-autonomous accumulation of tubulin was observed in Src-transformed cells (Supplementary Fig. S3a). In addition, intermediate filament protein keratin5 + 8 were also accumulated in the apical region of RasV12 cells that were surrounded by normal cells (Supplementary Fig. S4a). Next, we examined the effect of nocodazole, an inhibitor of microtubule polymerization. Nocodazole strongly disrupted the structure of microtubule filaments (Fig. 3b). Nocodazole also attenuated the frequency of plectin accumulation in RasV12- or Src-transformed cells surrounded by normal cells, whereas cytochalasin D, an inhibitor of actin polymerization, had no significant effect (Fig. 3b,c and Supplementary Fig. S3b,c). In addition, nocodazole diminished accumulation of keratin5 + 8 (data not shown), suggesting the coordinated regulation of these cytoskeletons. Furthermore, treatment of nocodazole substantially suppressed apical extrusion of RasV12 or Src cells (Fig. 3d and Supplementary Fig. S3d), suggesting a crucial role of microtubules in this process. Moreover, we showed that knockdown of plectin profoundly diminished accumulation of tubulin and keratin in RasV12-transformed cells surrounded by normal cells (Fig. 3e,f and Supplementary Fig. S4a,b). Collectively, these data demonstrate that plectin and microtubules mutually affect their accumulation, thereby promoting elimination of transformed cells from epithelia.

### Plectin positively regulates apical elimination of RasV12-transformed cells in concert with EPLIN and microtubules

**Figure 4:**
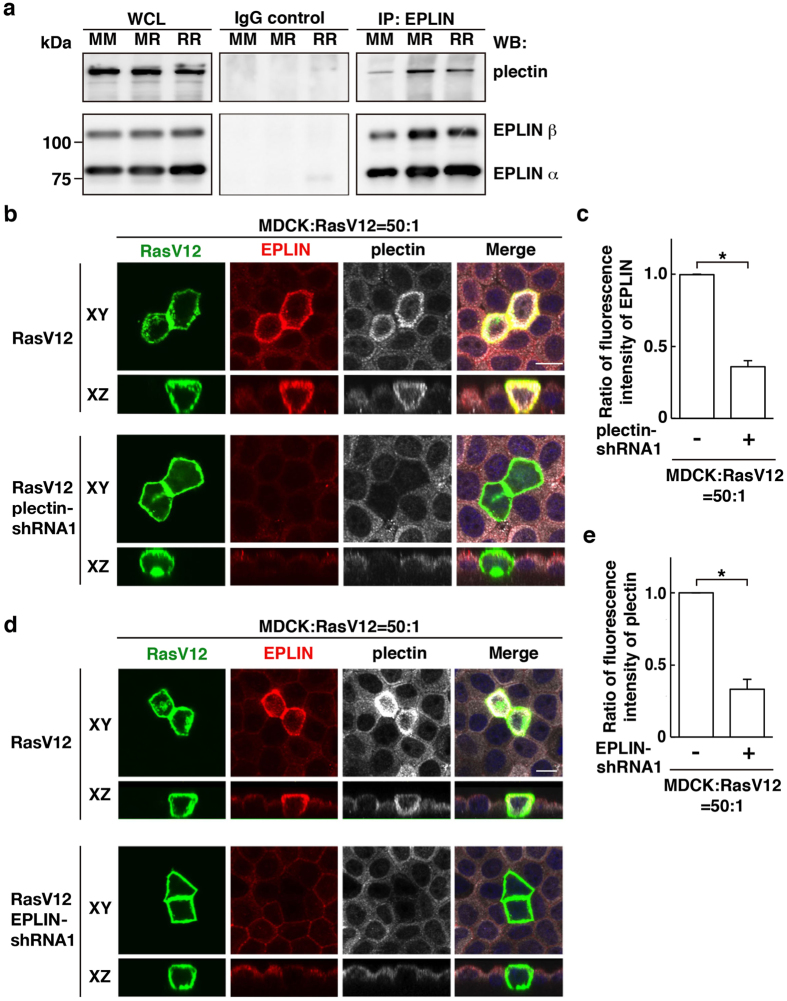
Plectin and EPLIN mutually regulate their accumulation in RasV12-transformed cells surrounded by normal epithelial cells. (**a**) Co-immunoprecipitation of EPLIN and plectin with anti-EPLIN antibody. MM, normal MDCK cells cultured alone; MR, 1:1 mix culture of normal MDCK and MDCK-pTR GFP-RasV12 cells; RR, MDCK-pTR GFP-RasV12 cells cultured alone. (**b**–**e**) Effect of plectin-knockdown on EPLIN accumulation (**b**,**c**) or effect of EPLIN-knockdown on plectin accumulation (**d**,**e**). MDCK-pTR GFP-RasV12 cells, MDCK-pTR GFP-RasV12 plectin-shRNA1 cells, or MDCK-pTR GFP-RasV12 EPLIN-shRNA1 cells were mixed with normal MDCK cells on collagen gels. Cells were fixed after 16 h incubation with tetracycline and stained with anti-EPLIN (red) and anti-plectin (white) antibodies and Hoechst (blue). (**b**,**d**) Scale bars, 10 μm. (**c**,**e**) Data are mean ± SD from three independent experiments. *P < 0.005; n = 30 cells for each experimental condition. Values are expressed as a ratio relative to plectin-shRNA1 (−) (**c**) or EPLIN-shRNA1 (−) (**e**).

**Figure 5:**
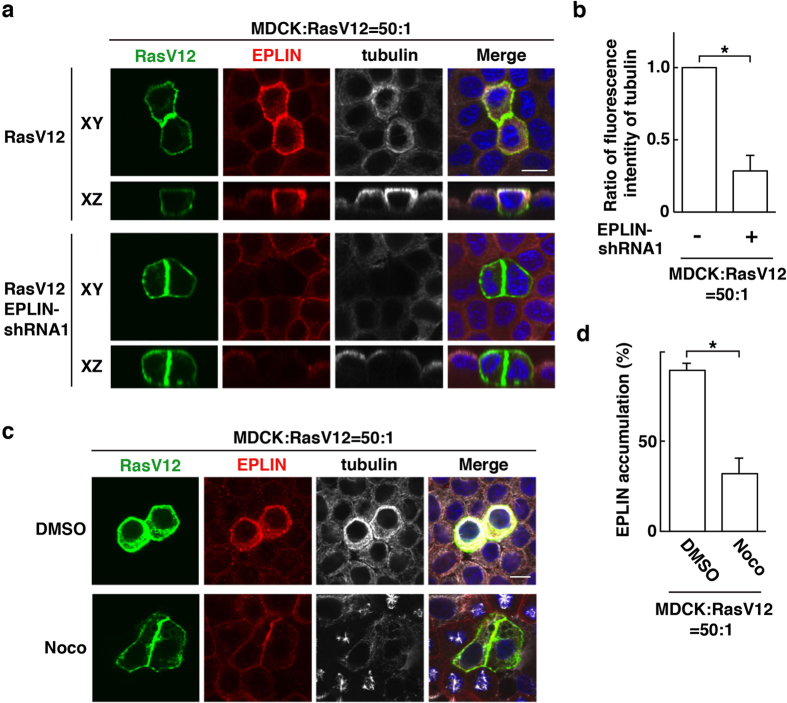
EPLIN and microtubules mutually regulate their accumulation in RasV12-transformed cells surrounded by normal epithelial cells. Effect of EPLIN-knockdown on tubulin accumulation (**a,b**) or effect of nocodazole on EPLIN accumulation (**c,d**). MDCK-pTR GFP-RasV12 cells or MDCK-pTR GFP-RasV12 EPLIN-shRNA1 cells were mixed with normal MDCK cells on collagen gels. Cells were fixed after 16 h incubation with tetracycline and stained with anti-EPLIN (red) and anti-tubulin (white) antibodies and Hoechst (blue). (**a,c**) Scale bars, 10 μm. (**b,d**) Data are mean ± SD from three independent experiments. (**b**) *P < 0.01; n = 30 cells for each experimental condition. Values are expressed as a ratio relative to EPLIN-shRNA1 (−). (**d**) *P < 0.0005; n = 100–110 cells for each experimental condition.

We have previously reported that EPLIN is accumulated in RasV12-transformed cells surrounded by normal cells and plays a vital role in apical extrusion of the transformed cells^14^. We thus examined the functional relevance between EPLIN and plectin. By immunoprecipitation, we found that plectin was co-immunoprecipitated with EPLIN under the mix culture condition of normal and RasV12-transformed cells (Fig. 4a). In addition, when plectin was knocked down in RasV12-transformed cells, EPLIN accumulation was significantly suppressed (Fig. 4b,c). Conversely, knockdown of EPLIN in RasV12 cells attenuated the accumulation of plectin (Fig. 4d,e). Similarly, EPLIN-knockdown suppressed tubulin accumulation, and treatment of nocodazole diminished EPLIN accumulation (Fig. 5a–d). When EPLIN was exogenously overexpressed within a monolayer of normal or RasV12 cells in a mosaic manner, accumulation of plectin or microtubules was not observed (Supplementary Fig. S5a,b). Collectively, these data indicate that plectin, EPLIN, and microtubules are co-accumulated in an interdependent manner in RasV12 cells surrounded by normal cells and that the interaction between normal and transformed cells is required for this process.

**Figure 6:**
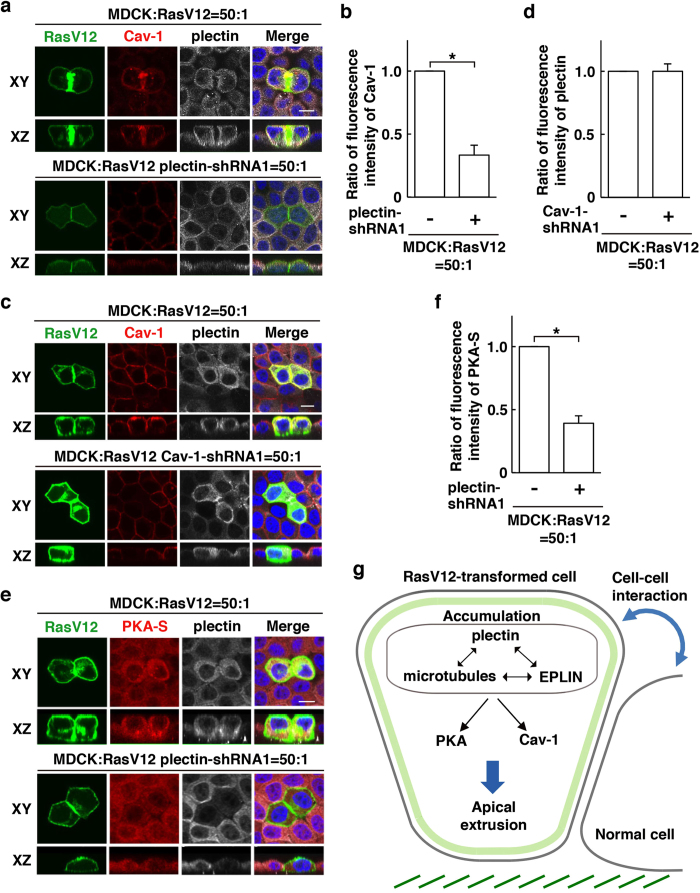
Plectin regulates Cav-1 enrichment and PKA activity in RasV12-transformed cells surrounded by normal epithelial cells. (**a**) Effect of plectin-knockdown on Cav-1 accumulation. (**c**) Effect of Cav-1-knockdown on plectin accumulation. (**e**) Effect of plectin-knockdown on activity of PKA. (**a**,**c**,**e**) MDCK-pTR GFP-RasV12 cells, MDCK-pTR GFP-RasV12 plectin-shRNA1 cells, or MDCK-pTR GFP-RasV12 Cav-1-shRNA1 cells were mixed with normal MDCK cells on collagen gels. Cells were fixed after 16 h incubation with tetracycline and stained with anti-plectin antibody (white) with anti-Cav-1 (red) or anti-PKA-substrate (red) antibody, and Hoechst (blue). Scale bars, 10 μm. (**b**,**d**,**f**) Quantification of (**a**,**c**,**e**). Data are mean ± SD from three independent experiments. n = 30 cells for each experimental condition. (**b**) *P < 0.0005, (**f**) *P < 0.005. Values are expressed as a ratio relative to plectin-shRNA1 (−) (**b**,**f**) or Cav-1-shRNA1 (−) (**d**). (**g**) The mode of action of the plectin-EPLIN-microtubules complex in apical extrusion of RasV12-transformed cells.

In the process of apical extrusion of RasV12-transformed cells, EPLIN functions upstream of Cav-1, a crucial component of caveolae, and regulates its enrichment in RasV12-transformed cells that are surrounded by normal cells^14^. In addition, EPLIN regulates activation of protein kinase A (PKA) that specifically occurs in RasV12 cells when they are surrounded by normal cells^14^. Plectin-knockdown diminished Cav-1 accumulation in RasV12-transformed cells surrounded by normal cells, whereas knockdown of Cav-1 did not affect accumulation of plectin (Fig. 6a–d). Furthermore, plectin-knockdown profoundly suppressed the non-cell-autonomous activation of PKA (Fig. 6e,f). Collectively, these data indicate that in concert with EPLIN and microtubules, plectin regulates these downstream regulators for apical extrusion of RasV12-transformed cells (Fig. 6g).

## Discussion

In this study, we have revealed that plectin is a crucial player in apical elimination of RasV12-transformed cells from the epithelium. Plectin is co-accumulated with EPLIN, microtubules, and intermediate filaments in RasV12 cells surrounded by normal cells and regulates downstream signaling molecules Cav-1 and PKA, thereby promoting apical extrusion (Fig. 6g). Plectin has been reported to interact with a variety of cytoskeletal proteins including three cytoskeletal filaments: actin, microtubule and intermediate filament^15^,^16^,^17^,^18^. Indeed, plectin is co-accumulated with tubulin and keratin in RasV12 cells surrounded by normal cells and is probably involved in the formation of integrated cytoskeletal webs. Previous reports have shown that plectin deficiency enhances the stability of microtubule filaments, suggesting that plectin mediates dynamic properties of microtubules^19^,^20^. As tubulin accumulation predominantly occurs at the apical membrane domain, it is plausible that accumulated plectin induces dynamic modulation of microtubules, which might provide physical forces and/or influence various cellular processes such as vesicle transport and membrane integrity that are required for apical extrusion. We have not observed the comparable accumulation of actin filaments^11^, suggesting that actin is not, at least, a major component within the cytoskeletal complex. EPLIN is also a crucial molecule in this complex, as knockdown of EPLIN substantially diminishes accumulation of plectin and microtubules. In this multiple-molecular complex, plectin seems to act as a key adaptor that interlinks these cytoskeletal proteins.

There still remains a question to be addressed: what are the upstream and downstream regulators of the plectin-EPLIN-microtubules complex? The accumulation of these molecules is mutually regulated; knockdown or disruption of one component diminishes the accumulation of the other. Thus, there should be a coordinated molecular machinery that induces the simultaneous assembly of these molecules in a concerted fashion. A previous study has demonstrated that filamin accumulates in normal epithelial cells at the interface with the neighboring transformed cells and that accumulated filamin promotes apical extrusion of the transformed cells^13^. Filamin crosslinks actin filaments to form orthogonal actin-meshworks^21^ and acts as mechanosensor/transducer^22^. Indeed, plectin can positively regulate cell stiffness and traction forces^23^. Thus, it is plausible that accumulated plectin modulates the physical properties of transformed cells, which then induces the recruitment of filamin in the neighboring normal cells. This is compatible with the recent reports demonstrating the involvement of physical forces in cell competition^24^,^25^,^26^. The mechanical link between filamin in normal cells and the plectin-EPLIN-microtubules complex in transformed cells needs to be elucidated in future studies. Recently, it has become clear that plectin, in addition to its role in cytoskeletal regulation, acts as a scaffold for signaling pathways by interacting with a number of signaling molecules including PIP2, Fer, RACK1, and AMPK^27^,^28^,^29^,^30^. Thus, it is plausible that plectin functions as a hub for the downstream signaling pathways in cell competition. In addition, the immunoprecipitation results in Fig. 1a,b suggest that there are unidentified, tyrosine-phosphorylated plectin-binding protein(s). To understand the missing link between the plectin complex and downstream molecules Cav-1 and PKA, we will need to identify binding protein(s) to the plectin-EPLIN-microtubules complex under the mix culture of normal and transformed cells.

Apical extrusion of transformed cells can be regarded as a cancer preventive process, which is supposed to occur at the initial stage of carcinogenesis. Thus, the plectin-EPLIN-microtubules complex could be a potential drug target; the activation of the plectin complex is expected to enhance the eradication of newly emerging and/or remaining transformed cells from epithelia. Further development of this study would open a new avenue for cancer preventive medicine.

## Materials and Methods

### Antibodies and Materials

Rabbit polyclonal affinity-purified anti-plectin antibody was generated using the following peptide as an antigen: CNKDTHDQLSEPSEVRSY (Medical & Biological Laboratories Co., Ltd). Rabbit anti-phospho-(Ser/Thr) PKA substrate and mouse anti-phospho-tyrosine (pY110, 9411S) antibodies were purchased from Cell Signaling Technology. Mouse anti-phospho-tyrosine (4G10) and mouse anti-α-actin (clone 4) antibodies were from Millipore. Mouse anti-GFP (a combination of two clones, 7.1 and 13.1) antibody was from Roche Diagnostics. Mouse anti-keratin K5/K8 (65131) antibody was from PROGEN Bio-technik GmbH. Rat monoclonal anti-α-tubulin (YOL1/34) and rabbit anti-caveolin (610059) antibodies were obtained from Abcam. Mouse anti-EPLIN antibody (sc-136399) was from Santa Cruz Biotechnology. Mouse anti-FLAG antibody (M2) was from Sigma-Aldrich. Alexa-Fluor-568- and -647-conjugated secondary antibodies were from ThermoFisher Scientific. Hoechst 33342 (Life Technologies) was used at a dilution of 1:5,000. For immunofluorescence, the primary antibodies describe above were diluted in PBS containing 1% BSA at 1:100, except anti-α-tubulin antibody at 1:200 and anti-EPLIN antibody at 1:50. All secondary antibodies were used at 1:200.

Alexa-Fluor-647-conjugated phalloidin (Life Technologies) was used at 1.0 U ml^−1^. For western blotting, primary antibodies were used at 1:1,000 except anti-α-actin antibody that was used at 1:2,000, and secondary antibodies were used at 1:1,000.

The following inhibitors were used where indicated: Cytochalasin D (Sigma-Aldrich, 100 nM) and nocodazole (Sigma-Aldrich, 200 ng ml^−1^). DMSO (Sigma-Aldrich) was added as a control.

### Cell Culture

MDCK and MDCK-pTR GFP-RasV12 cells were cultured as previously described^11^. ts-Src MDCK cells were cultured as described^12^. MDCK-pTR GFP-RasV12 cells stably expressing EPLIN-shRNA and MDCK-pTR GFP-cSrcY527F cells were established as described^14^. For Src-transformed cells, MDCK-pTR GFP-cSrcY527F cells were used except Supplementary Fig. S1a where ts-Src MDCK cells were used. MDCK-pTR GFP-RasV12 cells stably expressing plectin-shRNA were established as follows:

Double-stranded DNA fragments coding plectin-shRNA sequences (plectin-shRNA1: 5′-GATCCCCGCTTCAACTGGTCACGTATTTCAAGAGAATACGTGACCAGTTGAAGCTTTTTC-3′ and

5′-TCGAGAAAAAGCTTCAACTGGTCACGTATTCTCTTGAAATACGTGACCAGTTGAAGCGGG-3′ or plectin-shRNA2:

5′-GATCCCCGCATGATCATCATCATCATTTCAAGAGAATGATGATGATGATCATGCTTTTTC-3′ and

5′-TCGAGAAAAAGCATGATCATCATCATCATTCTCTTGAAATGATGATGATGATCATGCGGG-3′)were inserted into the *Bgl*II and *Xho*I site of pSUPER.neo+gfp (Oligoengine). MDCK-pTR GFP-RasV12 cells were transfected with pSUPER.neo+gfp plectin-shRNA using Lipofectamine 2000 (Invitrogen), followed by antibiotic selection in the medium containing 5 μg ml^−1^ blasticidin (InvivoGen), 400 μg ml^−1^ zeocin (InvivoGen), and 800 μg ml^−1^ G418 (Life Technologies).

To induce the expression of GFP-RasV12 or GFP-cSrcY527F in the tetracycline-inducible MDCK-pTR GFP-RasV12 or MDCK-pTR GFP-cSrcY527F cell lines, 2 μg ml^−1^ tetracycline (Sigma-Aldrich) was added. To transiently express EPLIN-FLAG, pcDNA3-EPLIN-FLAG was constructed as follows. The cDNA of mouse EPLINβ was inserted into the EcoRV and HindIII site of pcDNA3, and then an oligo fragment of FLAG was inserted into the SalI site. MDCK cells or MDCK-pTR GFP-RasV12 cells were transfected with pcDNA3-EPLIN-FLAG. At 6 h, media were changed to fresh media containing tetracycline, and at 24 h, cells were fixed and analyzed. For the inhibitor treatment, the indicated inhibitor was added together with tetracycline, and then cells were cultured with them for 16 h or 24 h. For immunofluorescence, cells were seeded onto Type-I collagen-mounted coverslips as previously described^11^,^12^.

### Immunofluorescence

MDCK-pTR GFP-RasV12 or MDCK-pTR GFP-cSrcY527F cells were mixed with MDCK cells at a ratio of 1:50 and cultured on the collagen matrix as previously described^11^. For immunofluorescence analyses, the mixture of cells was incubated for 8–12 h until they formed a monolayer, followed by tetracycline treatment for 16 h. Cells were fixed with 4% paraformaldehyde in PBS and permeabilized with 0.5% Triton X-100 in PBS, followed by blocking with 1% BSA in PBS. Primary or secondary antibody was incubated for 2 h or 1 h respectively at ambient temperature. After incubation with antibodies, immunofluorescence images were acquired using the Olympus FV1000 system and Olympus FV10-ASW software. For quantification of immunofluorescence intensity, 30 transformed cells were analyzed for each experiment using the MetaMorph software (Molecular Devices). For Fig. 6a,c,e, Alexa-Fluor-568-conjugated anti-Cav-1 or anti-PKA substrate antibody was prepared using Zenon^®^ Rabbit IgG Labeling Kit (Life Technologies) according to the manufacturer’s instructions. For analyses of apical extrusions, the samples were prepared as described above except that cells were treated with tetracycline for 24 h. More than 100 cells were analyzed for each experiment, and apically extruded cells were quantified.

### Immunoprecipitation and Western Blotting

MDCK and MDCK-pTR GFP-RasV12 or ts-Src MDCK cells were seeded at the density of 1.2 × 10^7^ cells in 14.5-cm dishes (two dishes for each experimental condition)(Greiner-Bio-One), and cultured at 37 °C for 6–8 h until a monolayer was formed. Tetracycline was then added to induce RasV12 expression. For immunoprecipitation, after 16 h culture with tetracycline, cells were washed with ice-cold PBS containing 1 mM Na_3_VO_4_, and lysed for 30 min in Triton X-100 lysis buffer (20 mM Tris-HCl [pH 7.5], 150 mM NaCl, and 1% Triton X-100) (for Fig. 1a,b and S1a) or RIPA lysis buffer (50 mM Tris-HCl [pH 8.0], 150 mM NaCl, 1% NP-40, 0.5% sodium deoxycholate, and 0.1% SDS) (for Fig. 4a) containing the following inhibitors: 1 mM Na_3_VO_4_, 10 mM NaF, 5 μg ml^−1^ leupeptin, 1 mM phenylmethylsulfonyl fluoride, and 7.2 trypsin inhibitor units of aprotinin. 0.1 mM Na_2_MoO_4_ was also added in Triton X-100 lysis buffer. The former and latter buffers were used for immunoprecipitation with anti-phospho-tyrosine and anti-EPLIN antibodies, respectively. After centrifugation of the cell lysates at 21,500 g for 10 min, the supernatant was first pre-cleared with sepharose protein G beads (GE Healthcare) for 30 min. The pre-cleared cell lysates were then incubated with control IgG-conjugated Dynabeads protein G (Life Technologies) for 30 min, and finally subjected to immunoprecipitation for 1 h with Dynabeads Protein G conjugated to anti-phospho-tyrosine antibodies (10 μg: a combination of 4G10 and pY110 at 1:1) or anti-EPLIN antibody (10 μg). Immunoprecipitated proteins were subjected to SDS-PAGE, followed by SYPRO Ruby protein gel staining (Life Technologies) or western blotting with the indicated antibodies. SYPRO Ruby protein staining was performed according to the manufacturer’s instructions. For Fig. 1a, the combination of immobilized phospho-tyrosine beads was used (Cell Signaling #9419 and Millipore #16–638). Stained gels and western blotting data were acquired using ImageQuant^TM^ LAS4010 (GE healthcare). Selected protein bands were identified by mass spectrometry as previously described^13^. To examine the plectin-knockdown efficiency, MDCK-pTR GFP-RasV12 cells stably expressing plectin-shRNA were seeded onto 6-cm dishes (Greiner-Bio-One) at 1 × 10^6^ cells. After 8 h, the cells were further incubated for 16 h in the absence or presence of tetracycline. The incubated cells were lysed with Triton X-100 lysis buffer containing protease inhibitors (5 μg ml^−1^ leupeptin, 1 mM phenylmethylsulfonyl fluoride, and 7.2 trypsin inhibitor units of aprotinin) and directly boiled with SDS-PAGE sample buffer (for Fig. 2a and Supplementary Fig. S2a). Western blotting was performed as described^11^. Full-length gels and blots are shown in Supplementary Fig. S6.

### Protein Identification by Mass-Spectrometry

Protein bands were excised from the gel, and the protein was identified by peptide mass fingerprinting (PMF) and post-source decay (PSD) MS/MS analysis. Briefly, protein bands were digested in-gel by trypsin gold (Promega) after reductive S-alkylation with dithiothreitol and iodoacetamide. After incubation at 37 °C for 12 h, tryptic peptides were purified by ZipTip mC18 (Millipore). High-purity alpha-cyano-4-hydroxycinnamic acid (Shimadzu GLC) was used as MALDI matrix. Mass spectrometric analysis was performed by AXIMA Performance MALDI-TOF/TOF MS (Shimadzu Corporation). The acquisition mass range was 700–4000 Da. Mass spectrometer was first externally calibrated by angiotensin II (*m/z* 1046.54) and ACTH fragment 18–39 (*m/z* 2465.20), and each result was internally calibrated by tryptic autolysis fragment (*m/z* 842.51 and 2211.10). The peak processing of peptide mass spectra and MS/MS ion selection were analysed by Launchpad version 2.9.1 (Shimadzu), and protein was identified against NCBI non-redundant canis familiaris database (2011_11, 16,245,521 sequence entries) on Mascot Server version 2.3 (Matrix Science), up to one missed cleavage, peptide tolerance of 0.2 Da, fixed modifications of cysteine carbamydomethylation, and variable modifications of methionine oxidation. MOWSE scores of PMF above 56 were considered significant (*P* < 0.05) and ion scores of MS/MS above 28 were indicated identity or extensive homology (*P* < 0.05).

### Data Analyses

Two-tailed Student’s *t*-tests were used to determine *P*-values for statistical analyses.

## Additional Information

**How to cite this article**: Kadeer, A. *et al*. Plectin is a novel regulator for apical extrusion of RasV12-transformed cells. *Sci. Rep.* **7**, 44328; doi: 10.1038/srep44328 (2017).

**Publisher's note:** Springer Nature remains neutral with regard to jurisdictional claims in published maps and institutional affiliations.

## Supplementary Material

Supplementary Fig. S1-5
