# Supplementary material for: Plectin is a novel regulator for apical extrusion of RasV12-transformed cells
Source: Sci Rep. 2017 Mar 10;7:44328. doi: 10.1038/srep44328 (PMC5345014; doi:10.1038/srep44328)
Supplement: Supplementary Fig. S1-5 [file srep44328-s1.pdf]

Supplementary Information

Plectin is a novel regulator for apical extrusion of RasV12-transformed cells

Ailijiang Kadeer, Takeshi Maruyama, Mihoko Kajita, Tomoko Morita, Ayana Sasaki, Atsuko Ohoka, Susumu Ishikawa, Masaya Ikegawa, Takashi Shimada, and Yasuyuki Fujita

Supplementary Figure S1-6

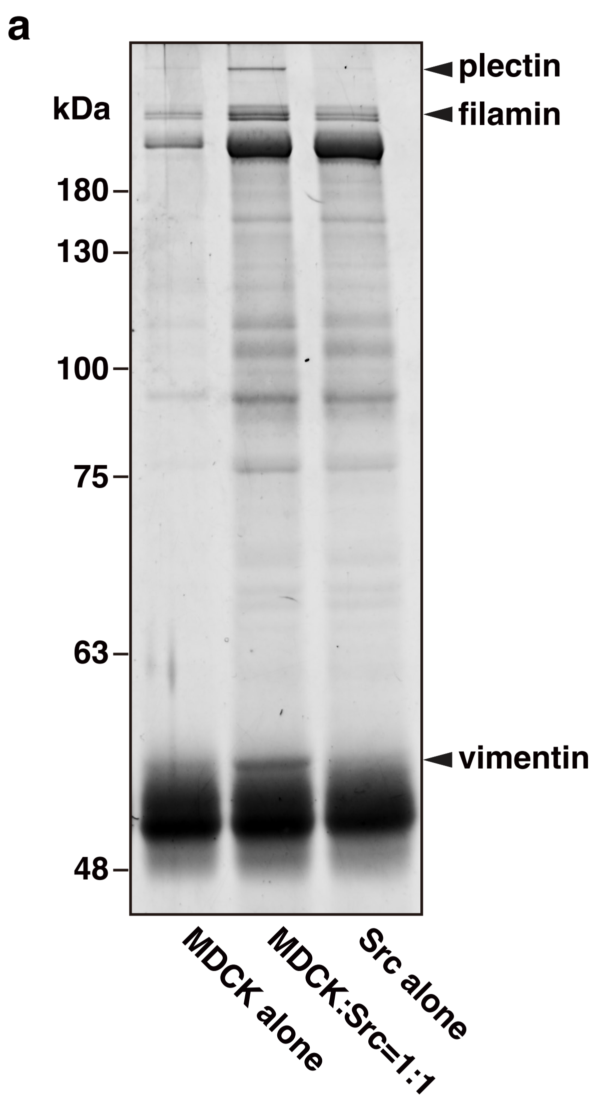

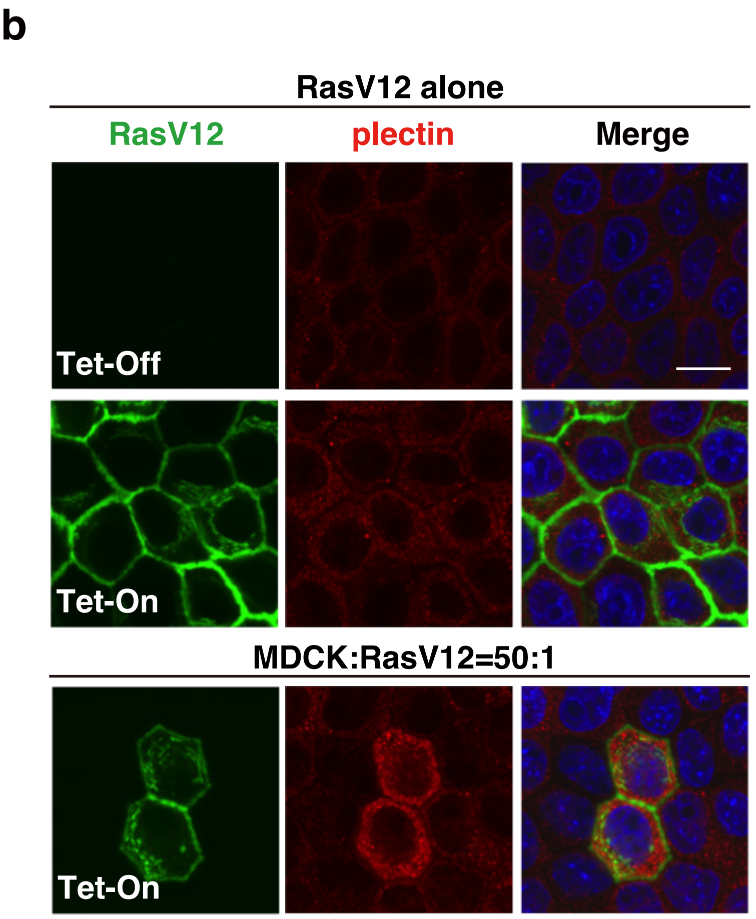

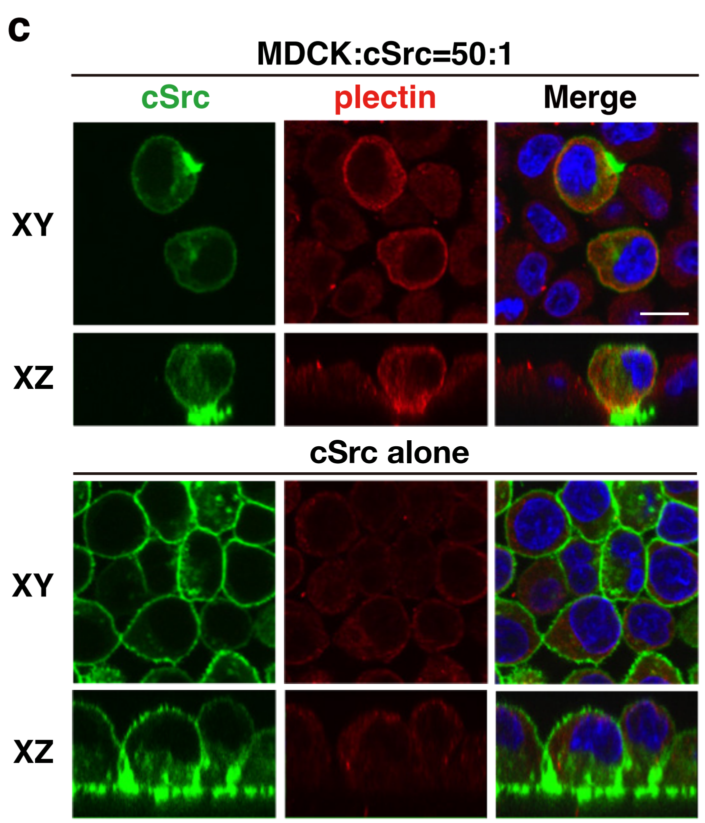

Figure S1. Plectin is accumulated in Src-transformed cells that are surrounded by normal epithelial cells. (**a**) SYPRO ruby staining (8% SDS-PAGE) of immunoprecipitated proteins with a mixture of anti-phospho-tyrosine antibodies. Cells were cultured under three different conditions: i) normal MDCK cells alone, ii) 1:1 mix culture of normal and Src-transformed MDCK cells, and iii) Src-transformed MDCK cells alone. Cell lysates were collected after 16 h incubation with the temperature shift, followed by immunoprecipitation. The arrowheads indicate the band for plectin, filamin, and vimentin. (**b**) Immunofluorescence images of plectin in RasV12-transformed MDCK cells that are cultured alone or co-cultured with normal MDCK cells in the presence or absence of tetracycline. MDCK-pTR GFP-RasV12 cells were mixed with normal MDCK cells or cultured alone on collagen gels. Cells were fixed after 16 h incubation with tetracycline and stained with anti-plectin antibody (red) and Hoechst (blue). Scale bar, 10 µm. (**c**) Immunofluorescence images of plectin in the mix culture of normal and Src-transformed MDCK cells. MDCK-pTR GFP-cSrcY527F cells were mixed with normal MDCK cells or cultured alone on collagen gels. Cells were fixed after 16 h incubation with tetracycline and stained with anti-plectin antibody (red) and Hoechst (blue). Scale bar, 10 µm.

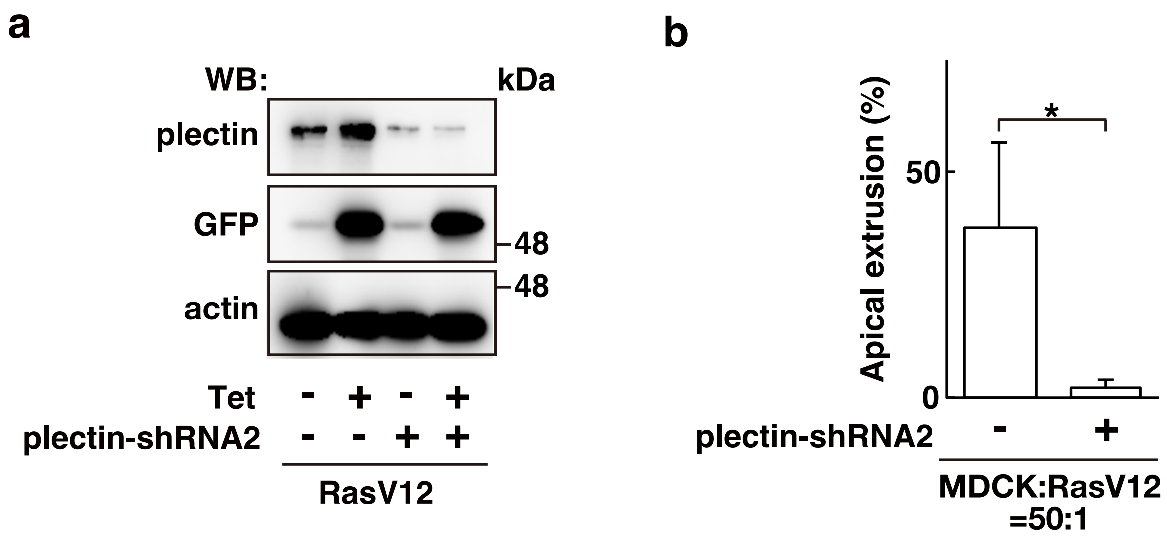

Figure S2. Plectin plays a positive role in apical extrusion of RasV12-transformed cells. (**a**) Establishment of MDCK-pTR GFP-RasV12 cells stably expressing plectin-shRNA2. Expression of GFP-RasV12 was induced with tetracycline treatment, followed by western blotting with the indicated antibodies. (**b**) Quantification of the effect of plectin-knockdown on apical extrusion of RasV12 cells. Data are mean ± SD from seven and three independent experiments. $^*P < 0.05$; n=100-290 cells for each experimental condition.

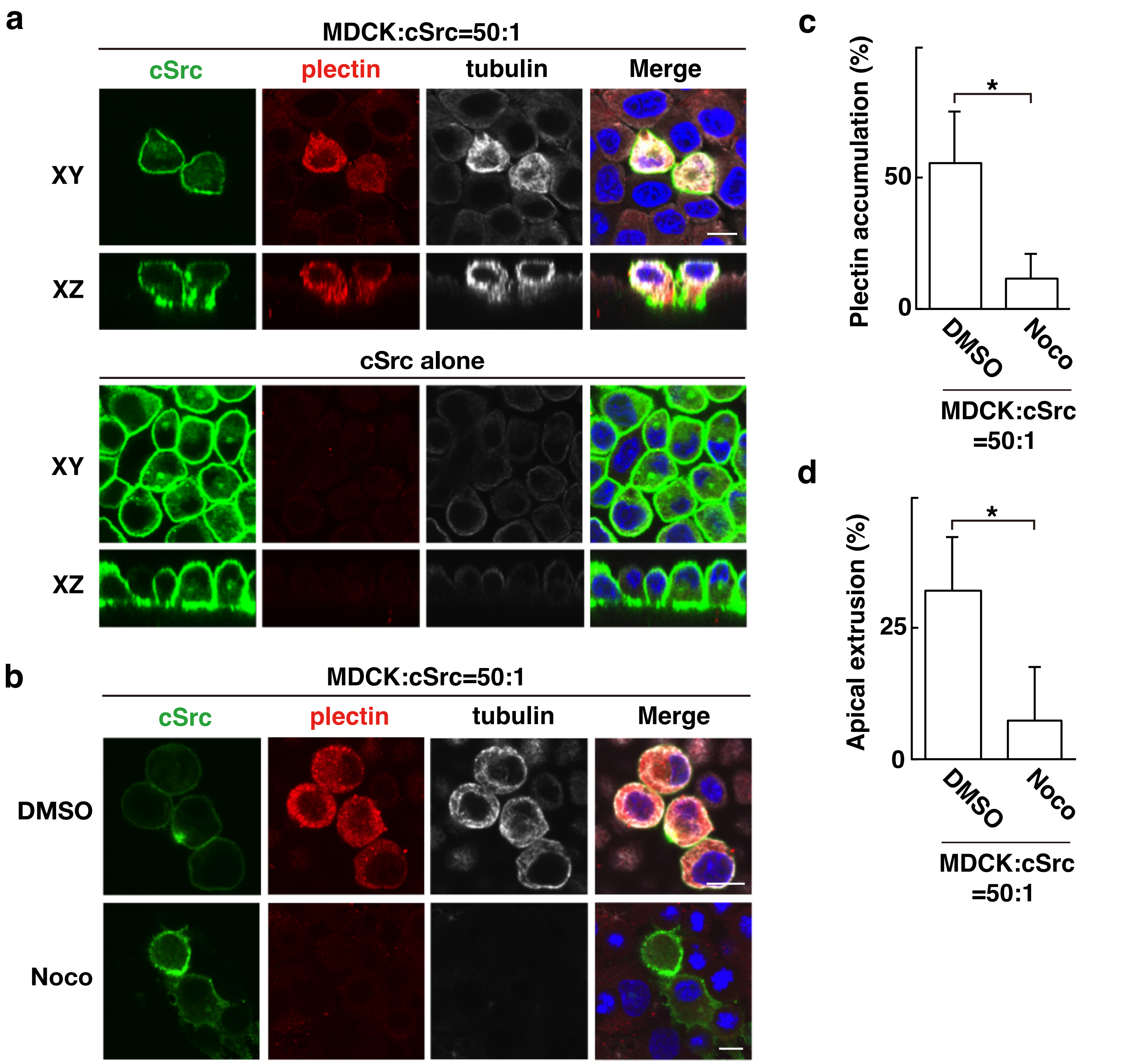

Figure S3. Microtubules and plectin co-regulate their accumulation and promote apical extrusion of Src-transformed cells. (**a**) Accumulation of plectin and tubulin in Src-transformed cells surrounded by normal epithelial cells. MDCK-pTR GFP-cSrcY527F cells were mixed with normal MDCK cells or cultured alone on collagen gels. Cells were fixed after 16 h incubation with tetracycline and stained with anti-plectin (red) and anti-tubulin (white) antibodies and Hoechst (blue). (**b**,**c**) Effect of nocodazole on accumulation of plectin and tubulin. (**c**) Data are mean ± SD from three independent experiments. *$P<0.05$; n=150-180 cells for each experimental condition. (**a**,**b**) Scale bars, 10 µm. (**d**) Effect of nocodazole on apical extrusion of Src-transformed cells. Data are mean ± SD from three independent experiments. *$P<0.05$; n=60-120 cells for each experimental condition.

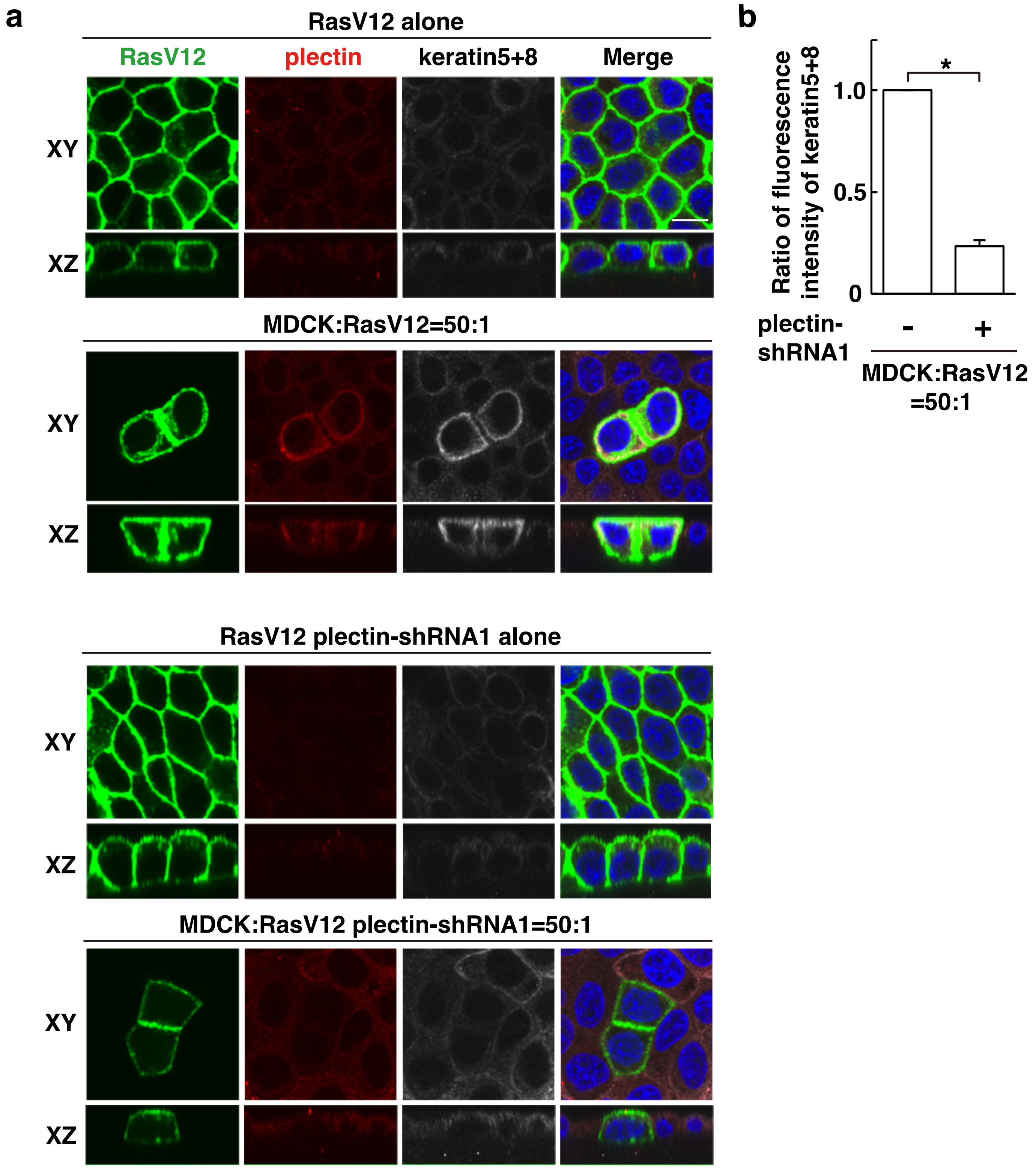

Figure S4. Plectin regulates accumulation of keratin intermediate filaments in RasV12-transformed cells surrounded by normal epithelial cells. (**a**) Accumulation of plectin and keratin5+8 in RasV12-transformed cells surrounded by normal epithelial cells. MDCK-pTR GFP-RasV12 cells or MDCK-pTR GFP-RasV12 plectin-shRNA1 cells were mixed with normal MDCK cells or cultured alone on collagen gels. Cells were fixed after 16 h incubation with tetracycline and stained with anti-plectin (red) and anti-keratin5+8 (white) antibodies and Hoechst (blue). (**b**) Effect of plectin-knockdown on accumulation of keratin5+8. Data are mean ± SD from three independent experiments. *P<0.001; n=30 cells for each experimental condition. Values are expressed as a ratio relative to plectin-shRNA1 (-).

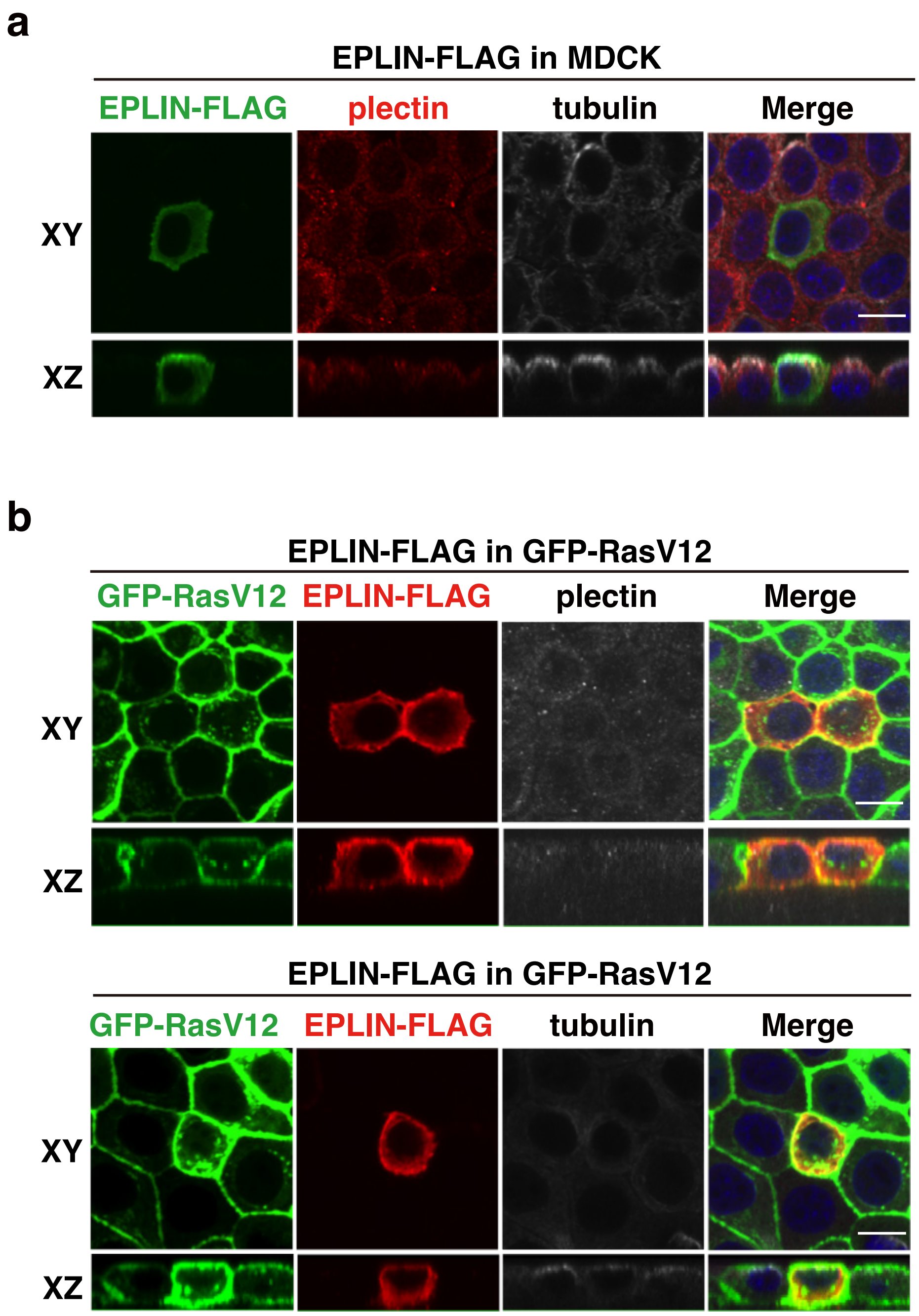

Figure S5. Overexpression of EPLIN alone is not sufficient to cause accumulation of plectin or microtubules. EPLIN-FLAG was transiently expressed within a monolayer of MDCK cells (**a**) or MDCK-pTR GFP-RasV12 cells (**b**) in a mosaic manner. Cells were fixed after 24 h of transfection and stained with anti-FLAG, anti-plectin, and/or anti-tubulin antibodies and Hoechst. Scale bars, 10 μm.

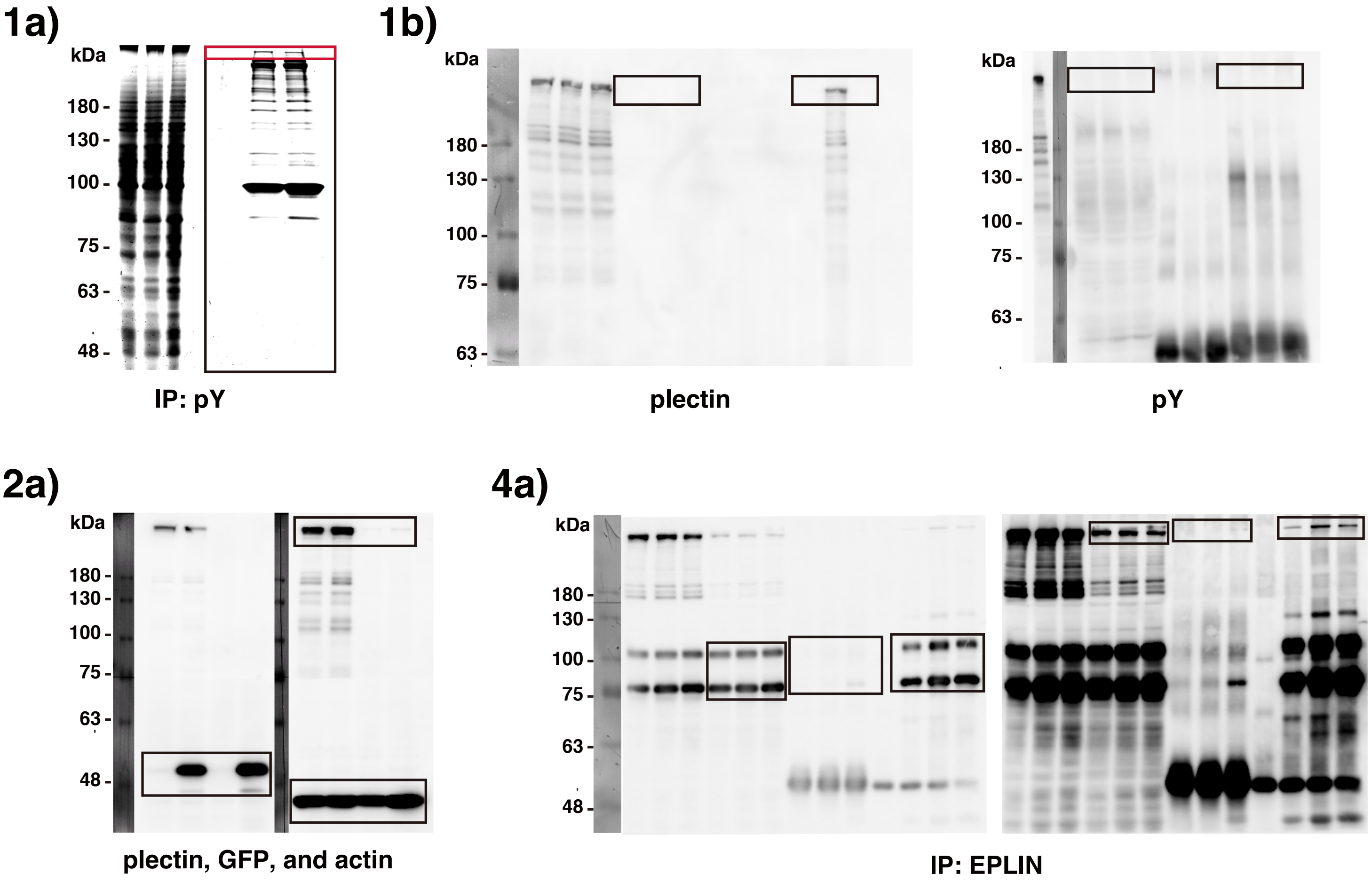

Figure S6. Full-length gels and blots
